# Habitual dietary protein intake affects body iron status in Japanese female college rhythmic gymnasts: a follow-up study

**DOI:** 10.1186/s40064-016-2569-7

**Published:** 2016-06-24

**Authors:** Yuki Kokubo, Kumiko Kisara, Yuri Yokoyama, Yoshiko Ohira-Akiyama, Yuki Tada, Azumi Hida, Sakuko Ishizaki, Yukari Kawano

**Affiliations:** Department of Health and Medical Science, Aichi Shukutoku University, 2-9 Katahira, Nagakute-shi, Aichi 480-1197 Japan; Department of Sport Wellness Science, Faculty of Sport and Health Sciences, Japan Women’s College of Physical Education, 8-19-1 KitaKarasuyama, Setagaya-ku, Tokyo, 157-8565 Japan; Graduate School of Agriculture, Tokyo University of Agriculture, 1-1-1Sakuragaoka, Setagaya-ku, Tokyo, 156-8502 Japan; Fujisangyo Co., Ltd., 5-32-7 shinbasi, Minato-ku, Tokyo, 105-0054 Japan; Department of Food and Nutritional Science, Faculty of Applied Bio-Science, Tokyo University of Agriculture, 1-1-1Sakuragaoka, Setagaya-ku, Tokyo, 156-8502 Japan

**Keywords:** Dietary protein intake, Iron deficiency, Female rhythmic gymnasts, Weight-loss period

## Abstract

**Background:**

Many rhythmic gymnasts stay lean by reducing their body weight (BW); however, this may result in iron deficiency (ID). Our previous cross-sectional study reported an association between ID incidence and protein intake in gymnasts during the pre-season. The present study aimed to examine the association between dietary protein intake and ID incidence in a 2-year follow-up study.

**Methods:**

Elite Japanese female college rhythmic gymnasts [mean age ± standard deviation (SD): 18.4 ± 0.5 years] were recruited on a voluntary basis every August for 9 years. Anthropometric, dietary intake, and hematological parameters were measured at baseline and 2 years later. A total of 20 participants without ID at baseline were divided into either a lower (L, n = 11) or higher (H, n = 9) protein group based on median protein intake (1.3 g protein/kg BW).

**Results:**

Participants consumed 1.08 ± 0.16 and 1.55 ± 0.14 g/kg BW of protein in the L and H groups, respectively. No significant changes in the intake of protein and other nutrients were observed between baseline and 2-year follow-up in both groups. ID was observed in a total of eight (72.8 %) participants in the L group and one (11.2 %) in the H group at follow-up. The incidence of ID was significantly lower in the H group than the L group (Fisher’s exact test, odds ratio, 0.043; 95 % CI 0.004–0.552; p = 0.010).

**Conclusions:**

During the pre-season weight loss period, habitually higher protein intake may reduce ID incidence among elite college female rhythmic gymnasts.

## Background

Rhythmic gymnasts compete with musical accompaniment using five different apparatus types, and are judged based on artistic beauty and performance, as well as speed, strength, and flexibility. In competitions, these gymnasts must perform physically demanding routines that last between 1.5 and 2.5 min. Their leanness and appearance greatly influence the performance of various movements. To maintain optimal sport-specific body size, body weight (BW), and body fat percentage (BF %) (Deutz et al. 2000; Soric et al. 2008), female rhythmic gymnasts sometimes strive to lose weight, with many practicing restrictive eating or skipping meals during the pre-season period (Cupisti et al. 2000).

Iron deficiency (ID) with or without anemia is the most widespread nutritional disorder, representing an important health issue for female athletes both in training and competition. Reduced hemoglobin causes changes in various physical capacities through decreased oxygen transport to active muscles, and decreased blood oxygen results in whole body fatigue and a feeling of weakness (Beard and Tobin 2000; Haas and Brownlie 2001). The prevalence of ID with or without anemia is between 11 and 38 % in female athletes (Malczewska et al. 2000; Beck et al. 2015; Dubnov and Constantini 2004), suggesting that ID incidence may vary with the demands of various sports. Recently, we conducted a cross-sectional study (Kokubo et al. 2016), and reported that ID occurs in 48.3 % of elite female rhythmic gymnasts during the pre-season weight-loss period. Furthermore, it was clear that rhythmic gymnasts showed a tendency toward insufficient nutrient intake and were at high risk for ID, particularly because of low protein intake (Kokubo et al. 2016). Dietary protein is a critical macronutrient for athletic performance to maintain lean body mass and repair muscle damage after strenuous exercise training. However, whether dietary protein intake affects subsequent body iron status in elite college female rhythmic gymnasts over the long term has yet to be clarified.

The aim of this study was to examine over a 2-year period the association between habitual dietary protein intake and body iron status during the pre-season weight loss period among Japanese elite female college rhythmic gymnasts.

## Methods

### Participants

Participants were a team of elite Japanese female college rhythmic gymnasts aged 18–19 years competing at the intercollegiate and/or national level, recruited every August for 9 years (2005–2013). All measurements were taken 1 or 2 weeks before the All Japan Intercollegiate Rhythmic Gymnastics Championship, which is held annually in August. Twenty-nine athletes underwent assessment twice: the first year of college (baseline) and the third year of college (follow-up). Nine participants had ID with or without anemia at baseline and were excluded from the study. A total of 20 participants [mean age ± standard deviation (SD)= 18.4 ± 0.5 years] were eligible for analysis with regard to physical, dietary, and hematological data. All participants lived alone in their own apartments. None were taking oral contraceptive pills, received any patient education, or were informed of the incidence of ID. All participants trained for approximately 6–8 h per day, 6 days per week before the competition in August at baseline and follow-up.

Informed consent forms describing the study objectives, benefits, and risks of participation were signed by all participants and their guardians. This study was approved by the Tokyo University of Agriculture ethics committee.

### Assessments

Anthropometric, dietary, and hematological data were collected 1 or 2 weeks before the competition at baseline and follow-up. Details on the standard methods of measurements and assessments have been described previously (Kokubo et al. 2016). On the measurement day, participants arrived at the study laboratory at 7:30 a.m. after 10 h of fasting. Anthropometric measurements (height, BW, BF %) and blood samples were taken, and habitual dietary intake was assessed via a face-to-face interview by a registered dietitian using a food frequency questionnaire (FFQ; Excel Eiyokun Data Systems, Kenpaku-sha, Japan) (Takahashi et al. 2001; Maruyama et al. 2015). Participants were also instructed to record the type, brand, and amount of iron supplements taken during the study period. Dietary analysis software (Excel Eiyokun Data Systems) was used to analyze habitual daily intake of food and nutrients. Blood samples were drawn using a standard venipuncture technique from the antecubital veins of participants. Tubes containing potassium EDTA were used to collect blood samples for the analysis of hematological parameters. Samples were analyzed by a local commercial laboratory (Medical Laboratory Systems Corp., Kanagawa, Japan). Height, BW, and BF % were measured in light clothing without shoes. Body mass index (BMI) was calculated as BW in kilograms (kg) divided by height in meters squared (m^2^).

### Criterion for body iron status

In this study, ID anemia was defined as hemoglobin (Hb) <12.0 g/dL, and ID was diagnosed if serum ferritin (SF) was <12.0 ng/mL (Gibson 2005; Peeling et al. 2007; Woolf et al. 2009).

### Statistical analysis

All data are expressed as mean ± SD or median. Participants were divided into two groups according to median protein intake adjusted for energy intake by residual analysis (g/kg BW); nine participants who consumed ≥1.30 g protein/kg BW were assigned to a higher protein group (H group), and the remaining 11 who consumed <1.30 g protein/kg BW to a lower protein group (L group). The Mann–Whitney U test was used to determine differences between the groups at baseline. Within-group changes at 2-year follow-up were tested with the Wilcoxon signed-rank test. Changes in various parameters from baseline to follow-up were adjusted for baseline data by the residual method, and differences in those changes between groups were compared by the Mann–Whitney U test. Fisher’s exact test was used to assess the relationship between ID incidence and H or L group, and to estimate odds ratios (ORs). p < 0.05 was considered statistically significant. All analyses were performed with IBM SPSS statistics for Windows (version 22, Japan).

## Results

### Participant characteristics

At baseline, mean dietary protein intake was 1.08 ± 0.16 g/kg BW in the L group and 1.55 ± 0.14 g/kg BW in the H group. There was no significant difference in habitual protein intake from baseline to follow-up between the groups. In addition, there was no significant difference in height, BW, BMI, or BF % between the groups at baseline. However, changes in BW, BMI, and BF % from baseline to follow-up were significantly smaller in the H group than in the L group (Table 1).

**Table 1:** Participant characteristics

	L group (n = 11)		H group (n = 9)	
	Baseline	Δ (baseline–follow-up)	Baseline	Δ (baseline–follow-up)
Protein (g/kg BW)	1.08 ± 0.16 (1.12)	0.00 ± 0.32 (0.02)	1.55 ± 0.14 (1.59)*	−0.11 ± 0.37 (−0.02)
Physical assessment				
Height (cm)	161.0 ± 3.3 (60.6)	0.7 ± 0.5 (0.7)	159.4 ± 2.8 (159.3)	0.3 ± 0.7 (0.2)
Body weight (kg)	48.0 ± 3.5 (47.4)	2.6 ± 2.6 (4.0)^†^	45.8 ± 3.7 (45.0)	0.4 ± 2.2 (1.0)*
Body mass index (kg/m^2^)	18.5 ± 0.9 (18.5)	0.9 ± 0.9 (1.4)	18.1 ± 1.4 (17.5)	0.0 ± 0.8 (0.2)*
Body fat (%)	14.6 ± 1.0 (14.3)	2.8 ± 2.2 (2.6)^†^	14.3 ± 2.9 (13.3)	0.4 ± 2.1 (0.4)*

Values are expressed as mean ± SD (median)

Protein intake was adjusted for daily energy intake by residuals analysis

Δ (baseline–follow-up): delta changes were adjusted for baseline values using the residual method

* p < 0.05; significantly different from the L group (Mann–Whitney U test)

^†^ p < 0.05; significantly different from baseline within-group (Wilcoxon Signed-rank test)

At baseline, the H group had significantly higher levels of protein per BW, protein, zinc, vitamin B_6_, and vitamin B_12_, as well as a higher animal protein ratio and significantly lower carbohydrate intake than the L group. In contrast, there was no significant difference between groups in habitual intake of energy, fat, total iron (diet with supplement), calcium and vitamin C. Moreover, the H group showed significantly higher habitual intake of cereals, green and yellow vegetables, fish and shellfish, meat and eggs, but significantly lower intake of confectioneries.

In the L group, habitual intake of zinc increased significantly at follow-up compared to baseline, and confectioneries decreased significantly. On the other hand, the H group showed no significant changes in habitual intake of any nutrient or food group between baseline and follow-up. Therefore, changes in intake of energy, all nutrients including protein, and food groups from baseline to follow-up were not statistically significant between groups (Table 2).

**Table 2:** Comparison of dietary intake

	L group (n = 11)		H group (n = 9)	
	Baseline	Δ (baseline–follow-up)	Baseline	Δ (baseline–follow-up)
Energy and nutrient intake				
Energy (kcal)	1873 ± 410 (1877)	121 ± 599 (48)	1941 ± 249 (1953)	29 ± 301 (25)
Protein (g)	51.6 ± 4.3 (53.5)	3.0 ± 16.7 (4.2)	70.4 ± 5.3 (71.4)*	−4.4 ± 17.4 (−1.6)
Animal protein (%)	40.1 ± 6.2 (38.4)	−2.3 ± 7.6 (0.3)	57.4 ± 8.5 (60.6)*	−3.9 ± 14.7 (−7.6)
Fat (g)	67.7 ± 7.5 (67.0)	0.7 ± 17.1 (−1.1)	66.7 ± 3.7 (66.7)	−2.4 ± 14.1 (−7.1)
Carbohydrate (g)	268 ± 20 (268)	27 ± 94 (10)	250 ± 9 (246)*	14 ± 28 (18)
Dietary iron (g)	7.6 ± 2.1 (7.6)	−0.4 ± 2.5 (−0.8)	7.0 ± 0.8 (7.1)	−0.2 ± 1.2 (−0.5)
Dietary iron with supplements (g)	9.0 ± 4.1 (7.6)	−2.6 ± 2.3 (−2.7)	10.9 ± 3.3 (12.9)	−1.3 ± 4.6 (−2.2)
Zinc (mg)	5.8 ± 0.5 (5.8)	0.5 ± 2.1 (0.3)^†^	8.1 ± 0.8 (7.8)*	−0.2 ± 1.9 (0.0)
Calcium (mg)	695 ± 169 (701)	8 ± 213 (27)	674 ± 98 (679)	39 ± 174 (72)
Vitamin B_6_ (mg)	0.60 ± 0.08 (0.58)	0.06 ± 0.26 (0.12)	0.94 ± 0.16 (0.97)*	−0.09 ± 0.27 (−0.18)
Vitamin B_12_ (mg)	2.8 ± 0.6 (2.7)	−0.7 ± 2.1 (−0.8)	7.5 ± 2.3 (8.1)*	−1.3 ± 4.1 (−3.2)
Vitamin C (mg)	74 ± 22 (78)	17 ± 31 (11)	67 ± 13 (61)	−7 ± 26 (−12)
Food groups				
Cereals (g)	231 ± 66 (218)	−5 ± 82 (−30)	341 ± 51 (338)*	23 ± 44 (30)
Green and yellow vegetables (g)	25 ± 10 (30)	10 ± 11 (11)	47 ± 18 (44)*	5 ± 23 (3)
Pulses (g)	39 ± 18 (39)	13 ± 32 (9)	63 ± 36 (53)	10 ± 49 (10)
Fish and shelfish (g)	17 ± 7 (19)	−14 ± 26 (−16)	79 ± 32 (83)*	−18 ± 50 (−49)
Meat (g)	46 ± 21 (43)	7 ± 29 (0)	70 ± 24 (85)*	−9 ± 20 (−6)
Eggs (g)	13 ± 7 (14)	8 ± 10 (9)	34 ± 16 (36)*	4 ± 15 (9)
Fruit (g)	100 ± 62 (108)	34 ± 91 (27)	76 ± 31 (60)	−19 ± 62 (−31)
Dairy products (g)	213 ± 72 (213)	−30 ± 82 (−53)	237 ± 100 (264)	10 ± 117 (13)
Confectioneries (g)	189 ± 51 (188)	−32 ± 81 (−75)^†^	89 ± 26 (96)*	1 ± 67 (11)

Values are expressed as mean ± SD (median)

Protein intake was adjusted for daily energy intake by residuals analysis

Δ (baseline–follow-up): delta changes were adjusted for baseline values using the residual method

* p < 0.05; significantly different from the L group (Mann–Whitney U test)

^†^ p < 0.05; significantly different from baseline within-group (Wilcoxon Signed-rank test)

### Hematological parameters

There were no significant differences between groups in any hematological parameters at baseline except for reticulocytes and SF; reticulocytes were significantly lower and SF significantly higher in the H group (Table 3). Only the L group showed significant within-group changes in SF, with SF significantly lower at follow-up compared to baseline. The only significant difference between groups in changes from baseline to follow-up was Ht; Ht levels increased in the H group, but decreased in the L group.

**Table 3:** Comparison of hematological parameters

	L group (n = 11)		H group (n = 9)	
	Baseline	Δ (baseline–follow-up)	Baseline	Δ (baseline–follow-up)
White blood cells (/μL)	5209 ± 1377 (4800)	−389 ± 996 (−872)	4800 ± 1236 (5200)	376 ± 1943 (−69)
Red blood cells (×10^4^/μL)	428 ± 38 (429)	−12 ± 22 (−9)	437 ± 39 (443)	−14 ± 20 (−17)
Hemoglobin (g/dL)	12.9 ± 0.5 (12.8)	−1.2 ± 1.3 (−0.5)	13.5 ± 0.9 (13.6)	−0.3 ± 0.5 (−0.2)
Hematocrit (%)	38.7 ± 2.0 (38.7)	−2.8 ± 3.8 (−2.6)	40.2 ± 3.4 (40.3)	0.5 ± 1.6 (0.8)*
MCV (fl)	91.2 ± 5.2 (90.0)	−3.3 ± 8.2 (−3.7)	92.3 ± 2.1 (92.3)	2.0 ± 4.5 (1.1)
MCH (pg)	30.5 ± 2.3 (30.3)	−1.7 ± 2.8 (−0.9)	30.9 ± 1.0 (30.8)	−0.1 ± 1.7 (−0.1)
MCHC (%)	33.4 ± 1.0 (33.4)	−0.9 ± 1.7 (−0.5)	33.5 ± 0.8 (33.4)	−0.7 ± 1.3 (−0.4)
Reticulocytes (%)	13.6 ± 6.0 (12.0)	−3.3 ± 3.1 (−4.9)	7.6 ± 5.6 (6.0)*	−3.2 ± 2.5 (−3.5)
Total iron binding capacity (μg/dL)	348 ± 62 (357)	9 ± 55 (19)	317 ± 55 (314)	−19 ± 50 (−8)
Serum free iron (μg/dL)	67.2 ± 37.9 (60.0)	1.7 ± 63.0 (−8.1)	94.6 ± 38.7 (93.0)	5.0 ± 40.2 (−1.1)
Transferrin saturation (%)	21.1 ± 15.5 (15.0)	1.1 ± 21.4 (−5.8)	29.9 ± 11.2 (32.6)	2.9 ± 17.1 (−0.6)
Serum ferritin (ng/mL)	26.5 ± 17.0 (16.0)	−21.0 ± 9.8 (−20.6)^†^	56.4 ± 29.5 (44.0)*	−16.3 ± 10.7 (−14.7)
Erythropoietin (mU/mL)	19.0 ± 5.3 (18.0)	15.0 ± 26.1 (6.4)	20.6 ± 4.4 (21.1)	0.6 ± 7.6 (1.8)
Haptoglobin (mg/dL)	25.4 ± 25.7 (11.0)	15.7 ± 30.6 (2.2)	30.7 ± 26.7 (22.0)	−4.9 ± 26.3 (−6.4)

Values are expressed as mean ± SD (median)

Δ (baseline–follow-up): delta changes were adjusted for baseline values using the residual method

*MCV* mean corpuscular volume, *MCH* mean corpuscular hemoglobin, *MCHC* mean corpuscular hemoglobin concentration

* p < 0.05; significantly different from the L group (Mann–Whitney U test)

^†^ p < 0.05; significantly different from baseline within-group (Wilcoxon Signed-rank test)

No participants had ID at baseline; however, four (36.4 %) participants had ID anemia and four (36.4 %) had ID in the L group at the 2-year follow-up. Thus, a total of eight (72.8 %) participants in the L group developed ID during the follow-up period. In contrast, no one developed ID anemia and only one (11.1 %) participant developed ID in the H group during the follow-up period (Table 4). The incidence ratio of ID with or without anemia was significantly lower in the H group than the L group (ORs = 0.043, 95 % CI 0.004–0.552; p = 0.010) (Table 4).

**Table 4:** Comparison of the incidence of iron deficiency (ID) at follow-up

Group	With ID	Without ID	*p*	ORs for ID incidence (95 % CI)
L (n = 11)	8 (72.8)	3 (27.2)	0.010	1 0.047 (0.004–0.552)
H (n = 9)	1 (11.2)	8 (88.8)		

Values represent numbers of participants (%). *p*: Fisher’s exact test

With ID: participants who developed iron deficiency; Without ID: participants who did not develop iron deficiency

Iron deficiency (ID) was defined as serum ferritin <12.0 ng/mL and/or Hb <12.0 g/dL

## Discussion

In this follow-up study, female collegiate rhythmic gymnasts were divided into L or H groups based on their habitual protein intake (median 1.30 g/kg BW) at the baseline assessment. As a result, habitual dietary protein intake remained consistent in both groups from baseline to follow-up, but the incidence of ID was significantly lower in the H group than the L group.

Participants in the H group habitually consumed more cereals, vegetables, animal-based foods, and more nutrients such as zinc, vitamin B_6_, and vitamin B_12_. Animal-based foods contain more protein than plant-based foods, as well as several nutrients including zinc, iron, vitamin B_6_ and vitamin B_12_ (Zanovec et al. 2010; Murphy et al. 2011; Nicklas et al. 2012). Snyder et al. (1989) previously reported that SF values are significantly lower in a vegetarian diet group than a red meat diet group, and suggested that dietary intake of heme–iron from animal-based food is associated with increased bioavailability of dietary iron. Reddy et al. (2006) reported that non-heme iron absorption increases with a high-protein diet from meat. Thane et al. (2003) also reported that consuming meat reduces the risk of poor iron status. In our study, SF levels at baseline were significantly higher in the H group than the L group, suggesting that participants in the H group at baseline already had a higher body iron status than the L group.

The Recommended Dietary Allowance (2015 RDA) for Japanese adult women (Ministry of Health, Labour and Welfare 2015) is 10.5 mg iron per day to prevent development of ID. In our present study, dietary iron intake with supplements did not meet the RDA for seven (63.6 %) and four (44.4 %) participants in the L and H groups, respectively, at baseline, and 10 (90.9 %) and seven (77.8 %) participants in the L and the H groups, respectively, at follow-up (data not show). These results show that many participants had insufficient total iron intake during the 2-year follow-up period. Woolf et al. (2009) reported that active women have lower body iron status than sedentary women despite having a greater total iron intake and similar heme iron intake. Spodaryk et al. (1996) showed that hematological indices are significantly lower in athletes when there is no difference in total dietary iron intake between athletes and sedentary women. These previous studies indicate that female rhythmic gymnasts are at higher risk of ID (Klentrou and Plyley 2003; Ishizaki et al. 2006; Kokubo et al. 2016), even if total iron intake is sufficient.

Body composition is an important performance factor in elite female rhythmic gymnasts. In our study, mean BW and BF % of the participants were 48.0 kg and 14.6 % in the L group, and 45.8 kg and 14.3 % in the H group, respectively. Maïmoun et al. (2013) and Georgopoulos et al. (2001) reported 52.4 ± 4.7 kg BW and 13.6 ± 3.6 BF %, and 45.3 ± 6.6 kg BW and 15.9 ± 4.9 BF %, respectively, in elite female rhythmic gymnasts. Our baseline data are consistent with these reports. In addition, it is clear that participants in the H group significantly attenuated increases in BW and BF % at 2-year follow-up compared with the L group. Recently, it was reported that higher protein intake helps lower BW maintenance during weight loss in athletes (Helms et al. 2014; Mettler et al. 2010). The present study did not estimate daily energy expenditures. Therefore, it is unknown whether or not habitually higher protein intake contributes to BW and/or BF % loss maintenance.

There are some limitations to this study. First, the sample size was small because there are few female elite college rhythmic gymnasts in Japan, and the participants were recruited over 9 years. This factor might have the greatest impact on the study observations. Second, we used the FFQ to estimate habitual dietary intake, but the validity of the FFQ to estimate the habitual dietary intake of athletes who are trying to maintain low body weight remains unclear. Third, we did not collect information about training volume and intensity. Ostojic and Ahmetovic (2008) reported that SF levels poorly correlate with training duration. Di Santolo et al. (2008) did not find any negative effects of exercise on iron stores in female athletes. However, there is no evidence that training volume and intensity did not result in any significant hematological changes. Fourth, not all participants had a regular menstrual cycle. Menstrual disorders are common in female athletes (Maïmoun et al. 2013), which could have affected ID incidence (Kim et al. 1993). There are few follow-up studies evaluating ID incidence in elite female athletes. Therefore, a strength of this follow-up study is the finding that habitually higher protein intake might lower ID incidence in elite female rhythmic gymnasts during the weight-loss period.

## Conclusions

Habitual dietary intake including protein was unchanged in the pre-season weight loss period from baseline to the 2-year follow-up assessment; higher protein intake from animal protein was related not only to intake of protein, but also of vitamins and minerals. Habitual protein intake might lower ID incidence among elite college female rhythmic gymnasts in Japan. Future studies will be needed to explore the mechanism of how habitual dietary intake attenuates incidence of ID in female rhythmic gymnasts or other female athletes who strive to lose weight.

## Abbreviations

ID: iron deficiency
BW: body weight
BF %: body fat percentage
BMI: body mass index
FFQ: food frequency questionnaire
ORs: odds ratio
CI: confidence interval
Hb: hemoglobin
Ht: hematocrit
SF: serum ferritin
MCH: mean corpuscular hemoglobin
MCV: mean corpuscular volume
MCHC: mean corpuscular hemoglobin concentration
